# Enhanced boxing punch impact with silicone cushioning

**DOI:** 10.3389/fspor.2024.1358224

**Published:** 2024-08-08

**Authors:** Sirichet Punthipayanon, Supranee Kwanboonchan, Pornthep Rachanavy, Chia-Hua Kuo

**Affiliations:** ^1^ Laboratory of Exercise Biochemistry, Institute of Sports Science, University of Taipei, Taipei, Taiwan; ^2^ Department of Sports Science, Srinakharinwirot University, Bangkok, Thailand; ^3^ Thailand Science Research and Innovation Office, Bangkok, Thailand; ^4^ School of Sports Science, Institute of Science, Suranaree University of Technology, Nakhon Ratchasima, Thailand; ^5^ School of Physical Education and Sports Science, Soochow University, Suzhou, China

**Keywords:** boxer, punching glove, elastic materials, International Boxing Association, force, biomechanical, combat sports, impact

## Abstract

**Introduction:**

Elastic cushioning materials protect human tissue from injury by absorbing impact energy and delaying its transfer. However, the potential compromise in force delivery to the hitting target remains unknown.

**Methods:**

To examine if silicone cushioning compromises punch force delivery to a hitting target, a double-blind crossover trial with 12 elite boxers was conducted following material tests. Each boxer delivered five maximal punches under two conditions: silicone-hand wrapping and gauze-hand wrapping, in counterbalanced order, with a 3-day interval between sessions. Force distribution along the Z-axis indicated the punch's intended direction, while forces along the X and Y axes represented force dissipation toward unwanted direction.

**Results:**

The material tests (based on ASTM International, West Conshohocken, PA, USA) demonstrated substantially higher compression to disruption for silicone than gauze of similar thickness. During the punching trials, the silicon-based hand wrapping exhibited slightly higher total force production (436 ± 33 N vs. 372 ± 12 N, *p* < 0.001) than the gauze-based hand wrapping. Moreover, force wastage, calculated as the sum of forces along the X and Y axes vs. the total force produced in percentage, was notably lower for silicone material (2.0% wastage) compared to gauze (3.8% wastage) (*p* < 0.001). The use of silicone materials lengthened the contact time between the punching fist and the hitting target from 35 ms to 50 ms (*p* < 0.001).

**Conclusion:**

The elastic cushion does not compromise the force delivery of the boxing glove to the hitting target. Instead, it appears to allow for additional maneuvering time for alignment during the fist-target contact with higher impact.

## Introduction

In boxing and combat sports, punching stands as a fundamental technique extensively employed in competition against opponents. Olympic boxers, on average, transfer around ∼17 Nm of energy with power ranging from ∼6,000 to 7,000 J/s to a wrist in a straight punch (1). Notably, wrist injuries in metacarpophalangeal and carpometacarpal joints are prevalent among boxers according to a longitudinal study of Great Britain's amateur boxing squad from 2005 to 2012 (2). Similar findings have been reported in other combat sports like martial arts (3). To bolster the safety and welfare of boxers, the International Boxing Association has authorized the use of gauze bandages for hand-wrapping during competitions.

It is surprising for the paucity of the study on force efficiency of cushion materials for boxers. Most of the research outcomes on materials as a human-used cushion comes from aesthetic purposes for touch pads (4), handicaps (5), and car industry (6). Cushions are often used to provide a layer of protection or padding (7). In boxing and martial arts, cushioning materials like foam or silicone might be suitable in hand wraps or gloves to absorb and redistribute the impact of punches, reducing the risk of injury to the hands and wrists. In addition, cushions are widely used for comfort against postural movement (8). This is widely applied in automotive and industrial settings to absorb shock or vibration (9). For example, in vehicles, cushions in seats or suspension systems help dampen vibrations from the road, enhancing ride comfort (10). However, these features cast an uncertainty on whether absorbed energy will be delivered to unintended direction for boxing purposes.

For boxers, delivering punch force accurately while safeguarding against wrist injuries is paramount. Using elastic cushioning to absorb biomechanical energy and delay its release offers a potential solution. However, there's uncertainty regarding whether employing such energy-buffering elastic materials in hand wraps might redirect force in unintended directions, leading to energy wastage during a boxing punch. Currently, there is a lack of research reporting on force delivery and energy wastage related to cushion materials for designing boxing gloves.

This study investigated the hypothesis that elite boxers experience reduced force transmission to the target when an elastic cushion is wrapped around their fists. In the first part of the present study, we aim to provide empirical data on silicone as the testing material for padding. We then conducted a double-blind counter-balanced crossover trial to determine biomechanical properties of the silicone-based hand wraps compared to conventional hand wraps.

## Methods

### Material test

The study examined the elasticity properties of the silicone and gauze cushion pad for hand wraps by subjecting them to a series of material tests (based on standardized methods of ASTM International, West Conshohocken, PA, USA) at Srinakharinwirot University's Department of Mechanical Engineering. Specimens were randomly selected from the same batch and divided into two groups for distinct tests.

The tensile properties were evaluated using the ASTM D412 Tensile Test for Rubber, specifically designed for determining vulcanized rubber's tensile properties. A Tensile Universal Testing Machine (Comtech QC-505 M1F, Taiwan), equipped with appropriate grips for specimen retention, was utilized. Dumbbell-shaped samples, prepared in accordance with ASTM D412 specifications, were securely mounted in the machine's grips and extended at a constant rate until rupture occurred. Parameters like strength and elongation at the maximum force were calculated following ASTM D412 standards (11, 12).

To assess the compression-deflection characteristics of the rubber, tests were conducted following ASTM D575 standards. A cylindrical specimen, meeting ASTM D575 specifications, was placed into a Universal Testing Machine (Comtech QC-501M1F, Taiwan, 300 kN). The machine applied a compressive force at a constant rate until the specimens reached the specified deflection point, enabling the evaluation of the material's ability to withstand compressive loads effectively (13).

The stress-strain of the gauze pad was determined using the ASTM Standard Test Method for Breaking Force and Elongation of Textile Fabrics ASTM D5035 (14, 15).

### Human trial of punching testing

In the trial, we assessed the proportion of force loss to unintended direction of boxing punches under the two types (gauze and silicone) of hand wrapping conditions by using a force plate as a hitting target. The force waste is presented by the percentage of force to unintended direction (X- and Y-axes) against the total force input (X-, Y-, and Z-axes) during boxing punches.

#### Participants

Twelve highly skilled young well-trained boxers, aged 17 ± 1 year with an average height of 166 ± 1 cm and a weight of 56 ± 3 kg, representing the National youth boxing team, were included in this study. Each participant boasted considerable experience competing at the highest echelons of international matches. Additionally, they each had a minimum of three consecutive years of experience at the national level and had represented the youth national team in international youth tournaments. These athletes underwent daily training at the Nakhon Ratchasima sports schools, designated as the national training camp. All participants were injury-free at the time of the data collection. This study was approved by the Ethics Committee of Suranaree University of Technology, Thailand (COA No. 59/2565). All the experiments were carried out in accordance with the declaration of Helsinki. Participants and their parents were fully informed of the objectives and risks of the study. Their parents or legal guardians signed an informed consent form before the study began.

### Protocol

At the first visit, participants were asked to avoid intense physical activity within the 24 h before the test measurements. A 10-min standard warmup was organized under the supervision of the sports scientist. Half of the boxer were randomized to either gauze-based hand wrapping or silicone-based hand wrapping. Both testing materials were concealed from the boxers by placing them inside black bags wrapped around the fist's surface. All subjects were blind to the material inside the bag. After the hand-wrapping process, the wrapped hand was inspected by the experienced coach for a similar wrapping technique between the 2 conditions.

### Punch test

The force delivery was assessed on a vertical 8218A force plate (Kistler, Instrumente AG, Winterthur, Switzerland) as the hitting target with a sampling rate of 1,000 Hz. A series of 14 mm markers were attached to the anatomical lower limb locations based on the plug-in-gait model to track the segmental motion during the punching performance test. An infrared camera system (Miqus M3 series, Qualisys, Göteborg, Sweden) consisting of eight cameras, recorded the trajectories of fifteen reflective markers mounted to the full body at 250 Hz. Participants were asked to punch as fast as possible when the light stimulus appeared, requiring the lead leg to step forward onto the force platform to jab punch, followed by a rapid cross punch to the target. The assessment was adjusted the displacement to the target according to the participant's arm distance to avoid leaning forward, increasing the force on the target. Participants performed 1 jab-cross force with 1-min rest between trials (16). All boxers performed 5 consecutive trials.

### Data analysis

Kinematic and kinetic data were filtered (low-pass) with a fourth-order zero-lag Butterworth filter with a cut-off frequency of 8 and 12 Hz, respectively. F_x_, F_y_ and F_z_ and punch speed were calculated from the Qualisys track manager (Qualisys, Göteborg, Sweden).

### Statistical analysis

The sample size required for significant observations was calculated in G ∗Power. At least 5 samples are necessary to achieve a power of 0.8 when assessing differences in force delivery along the Z-axis due to treatment. Relevant data was imported into SPSS 24.0 for statistical analysis. All data were subjected to the single-sample Kolmogorov–Smirnov (K-S) test to verify whether they obey the normal distribution. Descriptive statistics are represented as mean ± standard deviation. The paired *t*-test was used to compare the differences between rear punch forces between silicone- and gauze-wrapped conditions (for parametric tests). The significance level for type 1 error was set at *p* < 0.01. Cohen's *d* was used to indicate effect size. The value of d was classified into small (<0.5), medium (0.5–0.7), and large effect (≥0.8).

## Results

### Elastic properties of the materials

**Figure 1:**
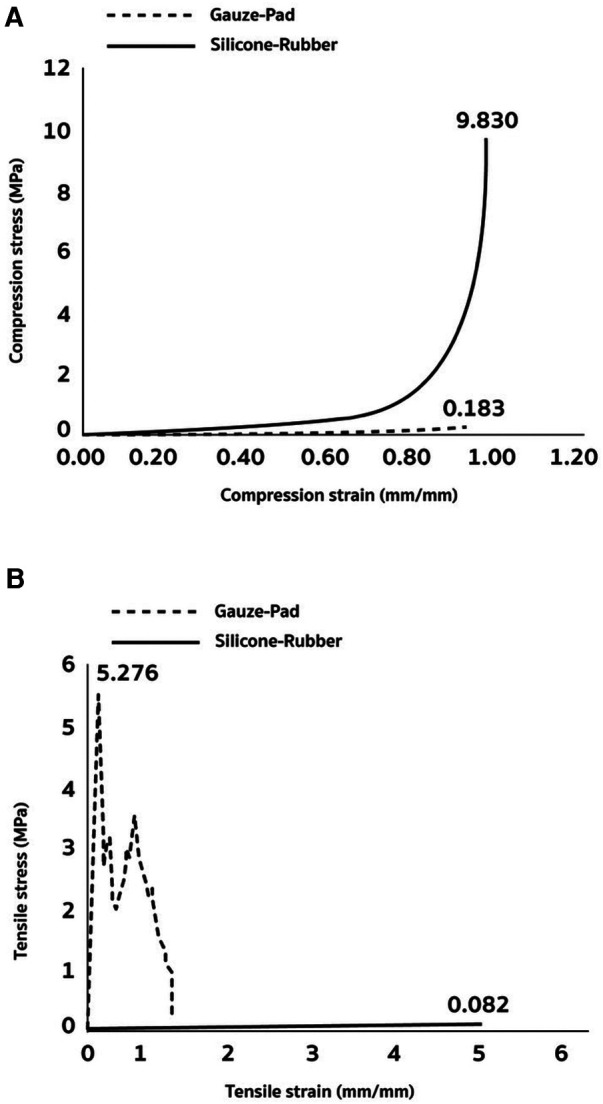
Material test outcomes of silicone cushion for boxing gloves. The silicone demonstrated a substantially greater tolerance to compression stress (**A**) and tensile stress (**B**) than gauze (the control material).

The elastic properties of silicone and gauze pads for hand wraps were evaluated through compression and tensile tests, involving the measurement of deformation under varying levels of pressure and tension. Figure 1 shows the outcomes of the tested materials, revealing notably distinct responses between the silicone and the gauze pad as a comparative reference material. In the compression test (Figure 1A), the silicone pad exhibited a maximum compression stress of 10.9 MPa at a compression strain point, and a compression strain of 0.973 mm/mm. Conversely, the gauze pad displayed a maximum compression stress of 0.9 MPa and a compression strain of 0.289 mm/mm. The large disparity between two materials highlights a substantial difference in elasticity (∼10 folds). Figure 1B illustrates the outcomes of the tensile test. The gauze pad exhibited a maximum tensile stress of 5.3 MPa and a tensile strain of 0.175 mm/mm before reaching the fracture threshold. Conversely, the silicone pad displayed a tensile stress of 0.08 mPa and a tensile strain of 5 mm/mm, reflecting an approximately fivefold difference in tensile strain when compared to the gauze pad.

### Human trials on force distribution

**Figure 2:**
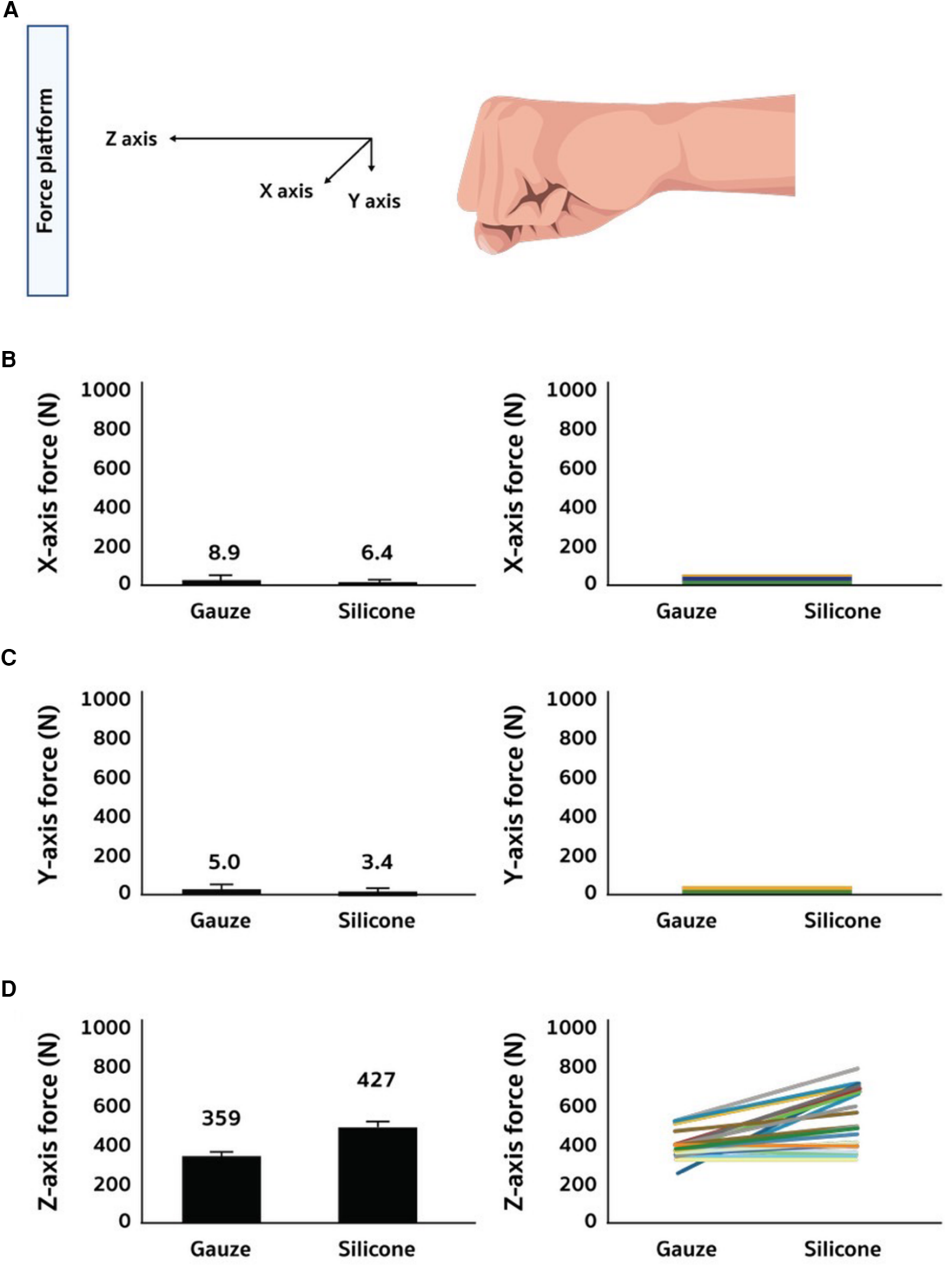
Force delivery to the hitting target of boxing punch. Punch force was measured by force plate at X-, Y-, and Z-axes (**A**). X- and Y-axes are regarded as unintended directions of punches (**B**,**C**). Z-axis is the intended direction to the punching target (**D**). Individual variations are shown on the right panel (**B**–**D**).

Table 1 shows the characteristic of boxers who participated in this double-blinded counter-balanced crossover trial. Most of them ranked top 100 in the world and 3 of them were gold medalists in the national championship. Figure 2 provides details of force production at 3-dimensions measured by force plate installed perpendicular against the boxing punching at two wrapped conditions, as depicted in Figure 1A. Figures 1B–D represents the force in newton (N) values during the 5 punches of 12 boxers measured at X-axis, Y-axis, and Z-axis. The average of pooled values is listed at the left panel and individual values are placed on the right panel. Silicon padded hand wrap decreased non-specific force exertion (force at X-axis, Y-axis) to the target (force plate) (*d* = 0.546, *p* = 0.002; *d* = 0.46, *p* = 0.001). Conversely, silicone padded hand wrap significantly increased Z-axis force exertion to the target than gauze-padded hand wrap (*d* = 0.65, *p* < 0.001).

**Table 1:** Characteristics of elite boxers.

Boxer	Weight (kg)	Height (m)	Experience (year)	National and World Ranking
S1	52	1.60	4	2nd IBA World Ranking
S2	56	1.72	3	22nd IBA World Ranking
S3	60	1.72	4	17th IBA World Ranking
S4	54	1.65	4	National Youth Championships Gold Medal
S5	58	1.73	4	22nd IBA World Ranking
S6	52	1.60	4	33rd IBA World Ranking
S7	56	1.63	4	National Youth Championships Gold Medal
S8	52	1.59	4	14th IBA World Ranking
S9	56	1.64	4	National Youth Championships Gold Medal
S10	60	1.70	4	21st IBA World Ranking
S11	52	1.60	4	35th IBA World Ranking
S12	58	1.75	4	74th IBA World Ranking

### Force wastage during boxing punches

**Figure 3:**
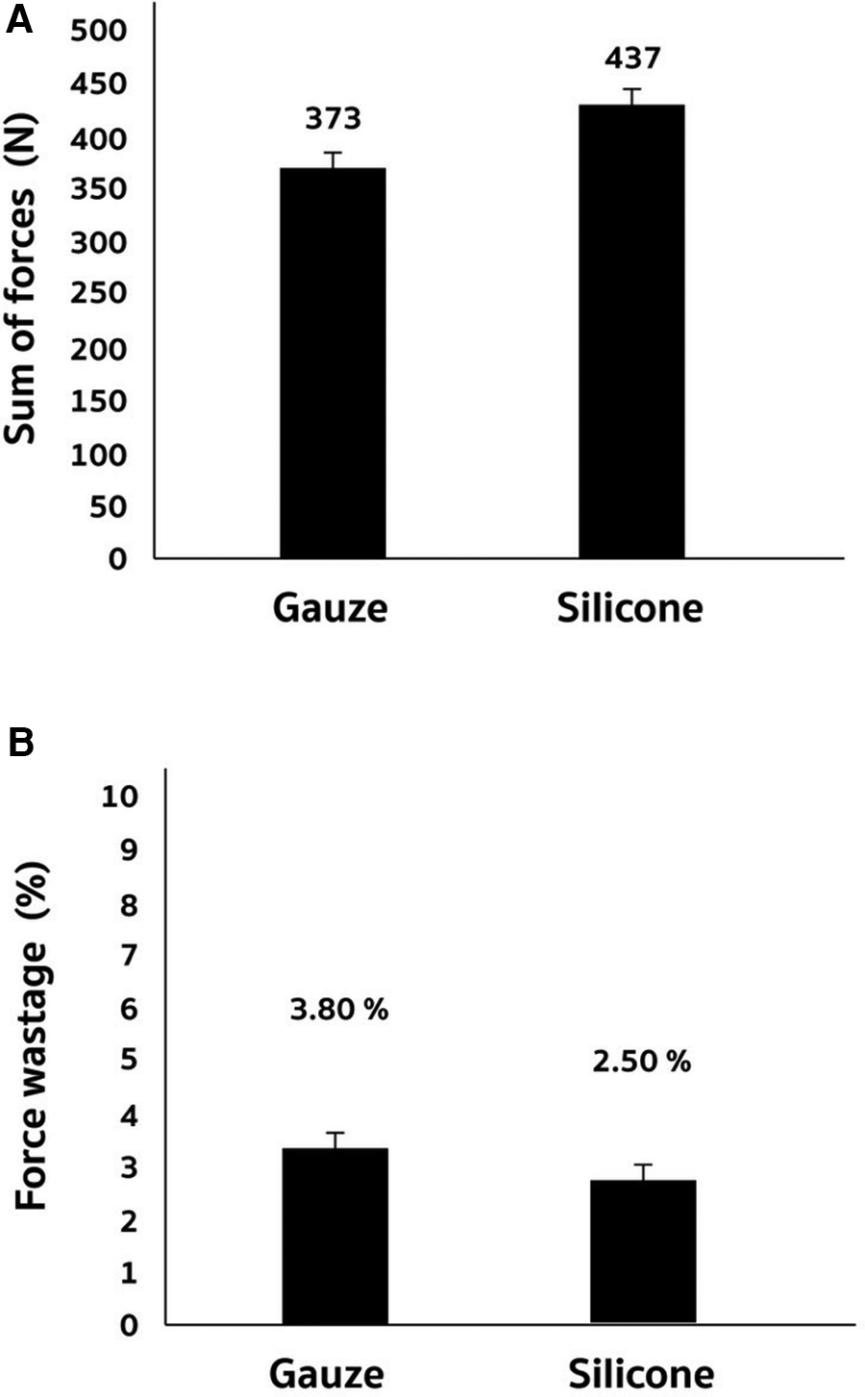
Force wastage of silicon-cushioned and gauze-cushioned punching fist of boxing glove to the hitting target. Total force is defined by the sum of force distributed to X-axis, Y-axis, and Z-axis (**A**). Force wastage is defined by percentage of the punching force distributed to unintended direction (sum of X-axis force and Y-axis force divide by total force in percentage) (**B**).

**Figure 4:**
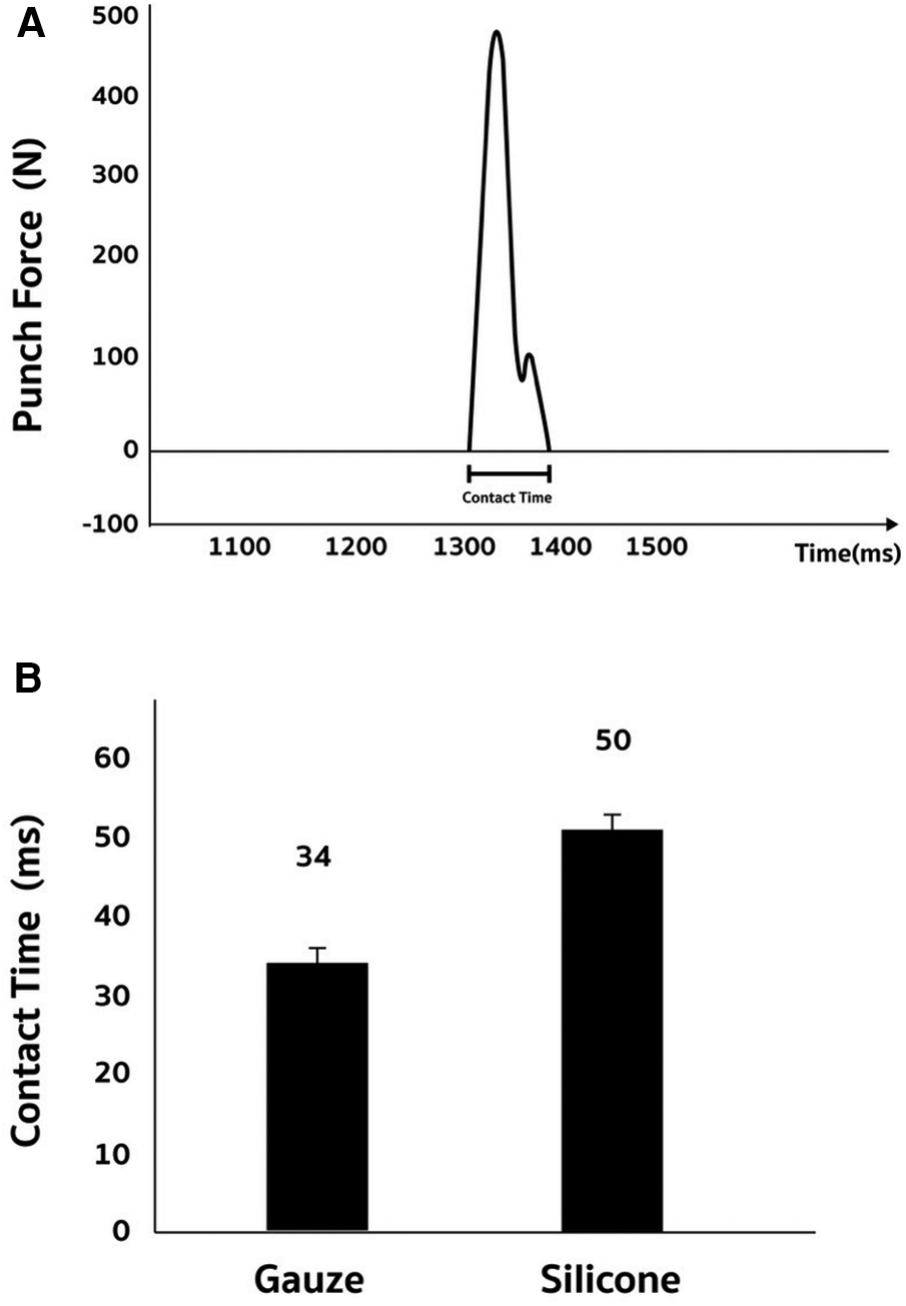
Average contact time between fist and target during boxing punches. A representative graph of a contact time recording on the force plate (**A**). Silicone increased contact time between fist and target (force plate) during boxing punches by the same boxers (**B**).

Based on the finding of non-target-specific force production (regarded as irrelevant force towards the punching target), we further calculate the total force in newton (N) and force wastage (%) of silicon-padded and gauze-padded punching to the hitting target (Figure 3). Both silicon-padded and gauze-padded punching conditions show similar total force output (sum of force from X-axis, Y-axis, and Z-axis) (Figure 3A). However, the exertional force of fist towards the target was enhanced by silicon-padded condition compared with gauze-padded condition, indicated by decreased percentage of force wastage (non-target-specific force production) to the total force output from a punch (Figure 3B). Figure 4 presents the contact time between the fist and the target (force plate) during a punch in boxing.

## Discussion

This study examined whether elastic cushioning compromises the punching force efficiency of boxers when directed towards a hitting target. The results from material test reveal significant distinctions in the mechanical property of the silicone and gauze pads under both compression and tension. Such variations suggest marked differences in elasticity and strength profiles between these materials. Force efficiency, defined as the percentage of force utilized in the intended direction during a punching task, was assessed in the human trial. The force plate utilized in this study enabled the measurement of force distribution towards the intended direction (Z-axis) and unintended directions (X-axis and Y-axis) for both types of cushioned hand wrapping. The results of the study demonstrate that the silicon-based hand wrap allows mildly greater total force directed towards the hitting target and a significantly higher force concentration specific to the target with reduced force wastage compared to the conventional gauze-based hand wrap. This key finding unveils an untapped advantage of employing elastic materials in combat sports. Furthermore, they suggest that materials with higher compressive properties, like silicon-based wraps, enable a more focused application of force towards the hitting target. This advantage may be linked to increased maneuverability associated with increased time of engagement between the fist and the hitting target.

Compared to the conventional gauze-based hand wrap, the extended contact duration facilitated by silicone-based hand wrapping offers a plausible explanation for the heightened force directed towards the target and the minimized loss of force in a boxing punch. As proprioception governs the adapted movement at high frequency in millisecond-scale (17), the increased contact time likely enables enhanced proprioceptive realignment along the intended direction (Z-axis) during a boxing punch at high velocity. Nerve impulses conduct at notably higher speeds (18, 19), and specifically, muscle spindle fibers responsible for proprioception transmit signals at velocities ranging from 72 to 120 m/s, dynamically responding to changes in muscle length and its rate of change (20). In the study, the peak velocity of a punch among 18-year-old boxers is only around 5 m/s, suggesting that silicone-based materials sufficiently allow for significantly extended contact time. This time extension during fist-target engagement potentially grants greater maneuverability to the punching motion, aiding its alignment with the intended direction during the contact between the fist and the target (21).

This study highlights the practical utility of elastic materials in enhancing force conservation when targeting impacts, complementing their established advantages in safety and protection. Through mechanical tests and human punching trials, a fresh perspective is offered, encouraging further exploration and refinement of elastic materials to amplify these benefits. The insights generated from this study could be pertinent to sophisticated target-oriented systems with robust computational capabilities. Advancements in high-speed feedback regulation within the neuromuscular system hold promise for industry applications related to energy conservation and precise target acquisition. Moreover, the development of highly elastic cushioning materials could emerge as a promising domain in engineering, extending beyond combat sports to areas such as humanoid robotics equipped with artificial intelligence. This could revolutionize grab-and-toss robotic systems, optimizing sensing-and-force output feedback loops for unparalleled precision.

Elastic cushioning materials play a crucial role in safeguarding human tissue by effectively absorbing energy from impacts (22). It is expected to delay the transfer of force to enhance overall protection (23). However, our data strongly indicates that integrating elastic cushioning into boxing gloves doesn't lead to force wastage. Rather, it enables improved punching techniques, aiding in the focused delivery of force towards the intended target. This advantage may be associated with increased time of fist-target engagement for force alignment during the joint maneuver. This novel discovery expands our understanding and opens new avenues for developing materials with higher elasticity. Such advancements hold the promise of not only optimizing force efficiency but also enhancing protective capabilities and comfort during combat sports. Embracing greater elasticity in these materials could revolutionize the benefits on force efficiency, protection, and overall user comfort.

The limitations of the study include the use of elite boxers as participants, raising questions about the transferability of the findings to novice boxers who are more prone to sports injuries. Additionally, it is unclear if female boxers produce similar outcome when using silicone cushion boxing gloves. Further research is necessary to address these uncertainties. The study design focuses solely on straight punches, warranting additional studies to validate the consistency of these results across different punching techniques. Moreover, the increased force delivery associated with silicone cushion boxing gloves should also be considered a potential risk factor for opponents during training.

## Data Availability

The raw data supporting the conclusions of this article will be made available by the authors, without undue reservation.
